# Identifying breast cancer patients at risk of relapse despite pathological complete response after neoadjuvant therapy

**DOI:** 10.1038/s41523-023-00525-2

**Published:** 2023-04-07

**Authors:** Jens Huober, Marion van Mackelenbergh, Andreas Schneeweiss, Fenja Seither, Jens-Uwe Blohmer, Carsten Denkert, Hans Tesch, Claus Hanusch, Christoph Salat, Kerstin Rhiem, Christine Solbach, Peter A. Fasching, Christian Jackisch, Mattea Reinisch, Bianca Lederer, Keyur Mehta, Theresa Link, Valentina Nekljudova, Sibylle Loibl, Michael Untch

**Affiliations:** 1 Universitätsfrauenklinik Ulm, Brustzentrum, Germany; 2 Kantonsspital St.Gallen, Brustzentrum, Departement Interdisziplinäre medizinische Dienste, St. Gallen, Switzerland; 3 Brustzentrum, Universitätsklinikum Schleswig-Holstein, Campus Kiel, Arnold-Heller-Straße 3, 24105 Kiel, Germany; 4 Nationales Centrum für Tumorerkrankungen, Heidelberg, Germany; 5 German Breast Group, Neu-Isenburg, Germany; 6 Gynäkologie mit Brustzentrum, Charité-Universitätsmedizin Berlin, Berlin, Germany; 7 Institut für Pathologie, Philipps-Universität Marburg, Marburg, Germany; 8 Centrum für Hämatologie und Onkologie Bethanien Frankfurt, Frankfurt, Germany; 9 Klinikum zum Roten Kreuz München, München, Germany; 10 Hämatologisch-Onkologische Schwerpunktpraxis Salat/Stötzer, München, Germany; 11 Zentrum Familiärer Brust- und Eierstockkrebs, Uniklinik Köln, Germany; 12 Universitätsklinikum Frankfurt, Frankfurt, Germany; 13 Universitätsklinikum Erlangen, Erlangen, Germany; 14 Sana Klinikum Offenbach, Offenbach, Germany; 15 Evang. Kliniken Essen-Mitte, Essen, Germany; 16 Department of Gynecology and Obstetrics, Technische Universität Dresden, Dresden, Germany; 17 HELIOS Klinikum Berlin Buch, Berlin, Germany

**Keywords:** Breast cancer, Outcomes research

## Abstract

This retrospective pooled analysis aims to identify factors predicting relapse despite a pathologic complete response (pCR) in patients with breast cancer (BC). 2066 patients with a pCR from five neoadjuvant GBG/AGO-B trials fulfill the inclusion criteria of this analysis. Primary endpoint is disease-free survival (DFS); secondary endpoints is distant DFS (DDFS) and overall survival (OS). After a median follow-up of 57.6 months, DFS is significantly worse for patients with positive lymph nodes (cN+ vs cN0 hazard ratio [HR] 1.94, 95%CI 1.48–2.54; *p* < 0.001). In patients with triple-negative tumors, lobular histology (lobular vs other HR 3.55, 95%CI 1.53–8.23; *p* = 0.003), and clinical nodal involvement (cN+ vs cN0 HR 2.45, 95%CI 1.59–3.79; *p* < 0.001) predict a higher risk of DFS events. Patients with HER2-positive cT3/4 tumors have a significantly higher risk of relapse (cT3/4 vs cT1 HR 2.07, 95%CI 1.06–4.03; *p* = 0.033). Initial tumor load and histological type predict relapse in patients with a pCR.

## Introduction

Neoadjuvant chemotherapy as part of a multimodal approach has been widely used for decades in patients with inflammatory, locally advanced, or inoperable breast cancer to achieve or facilitate operability and increase both local and systemic control^1^. In operable breast cancer, a recently published meta-analysis by the EBCTCG demonstrated that neoadjuvant chemotherapy was as effective as adjuvant chemotherapy in reducing the risk of distant recurrence and death from breast cancer. The rates of breast-conserving surgery were increased with the neoadjuvant approach and clinical response was highest in patients with HER2-positive and triple-negative breast cancer (TNBC)^2^.

Several individual trials and two patient-level meta-analyses showed that a pathological complete response (pCR) after neoadjuvant therapy is associated with improved event-free and overall survival^3–5^. The magnitude of this effect varied by molecular subtype and was most likely seen in patients with HER2-positive or triple-negative disease but also some luminal B-like tumors. In contrast, the long-term outcome of patients without a pCR is generally poor. Two recent randomized trials demonstrated that in these patients adding additional treatment after surgery - in case of TNBC capecitabine and in case of HER2-positive breast cancer TDM-1 - improved disease-free survival (DFS) and/or overall survival^6,7^. Although pCR rates have significantly increased with the implementation of targeted treatments and patients with pCR have a better long-term outcome than those without pCR, still 15–20% of patients with a pCR will relapse within the first 5 years. A better understanding of which patients will relapse despite a pCR may guide further treatment following surgery especially in patients with more aggressive tumors (like triple-negative or HER2-positive disease). Thus, we aimed to identify factors predicting relapse in patients with a pCR.

The presented study is a pooled analysis of the five neoadjuvant GBG/AGO-B trials GeparTrio, GeparQuattro, GeparQuinto, GeparSixto, and GeparSepto^8–21^ investigating factors predicting a relapse despite a pCR in the entire cohort and in subgroups according to tumor types.

## Results

### Patient characteristics

Between 2002 and 2013, a total of 7933 patients were recruited within the GeparTrio, GeparQuattro, GeparQuinto, GeparSixto, and GeparSepto trial, of whom 2066 (26%) had a pCR. The median age in patients with a pCR was 48 (range 21–75) years, 55% (*n* = 1125) were cN0 and 19% (*n* = 385) had a more advanced cT3/4 tumor at diagnosis. Most patients had a non-lobular histological tumor type (97%, *n* = 1997) while 40% (*n* = 805) and 39% (*n* = 780) of the patients had TNBC or HER2-positive disease, respectively. 1,41 (62%) patients had a high-grade tumor (G3) (Table 1).

**Table 1:** Patient, tumor and treatment characteristics.

Parameter	Category	GeparTrio *N* = 298	GeparQuattro *N* = 370	GeparQuinto *N* = 636	GeparSixto *N* = 296	GeparSepto *N* = 466	All patients *N* = 2066
		N (valid %)	N (valid %)	N (valid %)	N (valid %)	N (valid %)	N (valid %)
Age, years	Median (min, max)	45 (25–74)	49 (22–70)	48 (21–74)	46 (21–73)	49 (22–75)	48 (21–75)
	<30	9 (3.0)	8 (2.2)	27 (4.2)	13 (4.4)	17 (3.6)	74 (3.6)
	30−40	80 (26.8)	49 (13.2)	91 (14.3)	55 (18.6)	68 (14.6)	343 (16.6)
	40−50	107 (35.9)	138 (37.3)	253 (39.8)	109 (36.8)	166 (35.6)	773 (37.4)
	50−60	64 (21.5)	106 (28.6)	175 (27.5)	82 (27.7)	120 (25.8)	547 (26.5)
	60−70	35 (11.7)	66 (17.8)	79 (12.4)	31 (10.5)	80 (17.2)	291 (14.1)
	70+	3 (1.0)	3 (0.8)	11 (1.7)	6 (2.0)	15 (3.2)	38 (1.8)
BMI	<25 kg/m^2^	169 (56.9)	196 (53.0)	321 (50.5)	166 (56.1)	243 (52.1)	1095 (53.0)
	25–<30 kg/m^2^	93 (31.3)	116 (31.4)	202 (31.8)	93 (31.4)	134 (28.8)	638 (30.9)
	≥30 kg/m^2^	35 (11.8)	58 (15.7)	113 (17.8)	37 (12.5)	89 (19.1)	332 (16.1)
	missing	1	0	0	0	0	1
Menopausal status	premenopausal	181 (61.1)	217 (58.6)	378 (60.1)	199 (67.2)	267 (57.3)	1242 (60.4)
	postmenopausal	115 (38.9)	153 (41.4)	251 (39.9)	97 (32.8)	199 (42.7)	815 (39.6)
	missing	2	0	7	0	0	9
cT	cT1	5 (1.7)	18 (4.9)	127 (20.0)	98 (33.3)	181 (39.0)	429 (20.8)
	cT2	228 (76.8)	275 (74.3)	359 (56.4)	156 (53.1)	229 (49.4)	1247 (60.5)
	cT3	42 (14.1)	32 (8.6)	78 (12.3)	29 (9.9)	28 (6.0)	209 (10.1)
	cT4	22 (7.4)	45 (12.2)	72 (11.3)	11 (3.7)	26 (5.6)	176 (8.5)
	missing	1	0	0	2	2	5
cN	cN0	145 (49.2)	162 (43.8)	318 (50.3)	186 (64.1)	314 (68.4)	1125 (55.0)
	cN1	137 (46.4)	189 (51.1)	275 (43.5)	89 (30.7)	136 (29.6)	826 (40.4)
	cN2	8 (2.7)	18 (4.9)	31 (4.9)	13 (4.5)	5 (1.1)	75 (3.7)
	cN3	5 (1.7)	1 (0.3)	8 (1.3)	2 (0.7)	4 (0.9)	20 (1.0)
	missing	3	0	4	6	7	20
ER/PgR	Both ER, PgR negative	177 (65.6)	215 (58.1)	396 (62.3)	236 (79.7)	191 (41.0)	1215 (59.6)
	ER and/or PgR positive	93 (34.4)	155 (41.9)	240 (37.7)	60 (20.3)	275 (59.0)	823 (40.4)
	missing	28	0	0	0	0	28
HER2 status	Negative	244 (100)	202 (54.6)	425 (66.8)	157 (53.0)	204 (43.8)	1232 (61.2)
	Positive	0 (0.0)	168 (45.4)	211 (33.2)	139 (47.0)	262 (56.2)	780 (38.8)
	missing	54	0	0	0	0	54
Biological subtype	HER2-/HR +	84 (36.4)	80 (21.6)	154 (24.2)	0 (0.0)	96 (20.6)	414 (20.7)
	TNBC	147 (63.6)	122 (33.0)	271 (42.6)	157 (53.0)	108 (23.2)	805 (40.3)
	HER2 + /HR +	0 (0.0)	75 (20.3)	86 (13.5)	60 (20.3)	179 (38.4)	400 (20.0)
	HER2 + /HR-	0 (0.0)	93 (25.1)	125 (19.7)	79 (26.7)	83 (17.8)	380 (19.0)
	missing	67	0	0	0	0	67
Tumor grading	Grade 1	5 (2.0)	6 (1.7)	5 (0.8)	5 (1.7)	7 (1.5)	28 (1.4)
	Grade 2	94 (36.7)	156 (44.2)	236 (37.3)	83 (28.0)	165 (35.4)	734 (36.6)
	Grade 3	157 (61.3)	191 (54.1)	391 (61.9)	208 (70.3)	294 (63.1)	1241 (62.0)
	missing	42	17	4	0	0	63
Histological tumor type	Lobular subtype	19 (6.4)	17 (4.6)	22 (3.5)	1 (0.3)	8 (1.7)	67 (3.2)
	Non-lobular	278 (93.6)	353 (95.4)	613 (96.5)	295 (99.7)	458 (98.3)	1997 (96.8)
	missing	1	0	1	0	0	2
Ki-67, %	Median (min, max)	46.5 (1.5−97.5)	40.0 (0-90.0)	40 (1.0-100)	40.0 (3.0−95.0)	40.0 (3.0−95.0)	40.0 (0.0−100)
	≤20%	25 (15.2)	22 (25.3)	44 (18.8)	52 (17.6)	101 (21.7)	244 (19.6)
	>20%	139 (84.8)	65 (74.7)	190 (81.2)	244 (82.4)	365 (78.3)	1003 (80.4)
	missing	134	283	402	0	0	819
Number of cycles of chemo-therapy scheduled	≤6	160 (53.7)	0 (0.0)	0 (0.0)	296 (100)	0 (0.0)	456 (22.1)
	>6	138 (46.3)	370 (100)	636 (100)	0 (0.0)	466 (100)	1610 (77.9)
Clinical response after 2–4 cycles	Complete response	87 (29.3)	61 (16.5)	118 (18.8)	40 (13.8)	93 (20.8)	399 (19.6)
	Partial response	195 (65.7)	270 (73.0)	471 (75.0)	203 (70.0)	288 (64.4)	1427 (70.2)
	Stable disease	15 (5.1)	39 (10.5)	38 (6.1)	42 (14.5)	51 (11.4)	185 (9.1)
	Progress	0 (0.0)	0 (0.0)	1 (0.2)	5 (1.7)	15 (3.4)	21 (1.0)
	missing	1	0	8	6	19	34
Residual CIS	ypT0	254 (85.2)	273 (73.8)	501 (78.8)	237 (80.1)	407 (87.3)	1672 (80.9)
	ypTis	44 (14.8)	97 (26.2)	135 (21.2)	59 (19.9)	59 (12.7)	394 (19.1)

Data are N and valid % unless otherwise stated; *BMI* body mass index, *CIS* carcinoma in situ, *ER* estrogen receptor, *PgR* progesterone receptor, *HER2* human epidermal growth factor receptor 2, *HR* hormone receptor, *TNBC* triple-negative breast cancer.

After a median follow-up of 58.0 months (IQR 47.0–73.6), 269 events for DFS, 184 for DDFS, and 118 for OS were observed. Survival curves showing patients at risk by one year increment are provided in Supplementary Figure 1a–c. Residual in situ disease was associated with a significantly worse DFS, but not DDFS or OS. Median follow-up for the most recent studies GeparSixto and GeparSepto was shorter (48.4 and 49.7 months, respectively), thus an estimation of 4-year survival rates was reported in the following.

### Association of potential risk factors and outcome

**Fig. 1:**
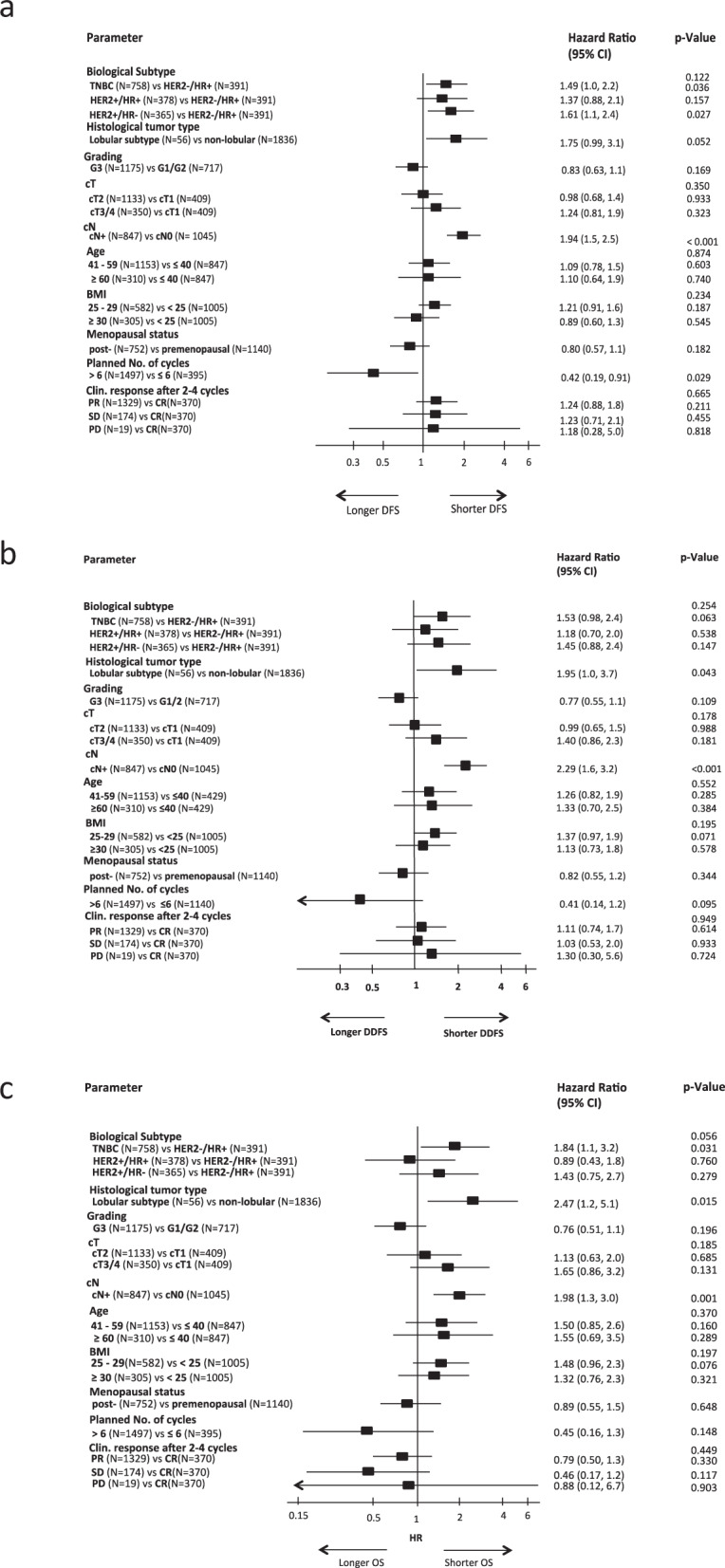
Multivariate Cox regression models for disease-free survival (a), distant disease-free survival (b) and overall survival (c) for the total population. Error bars represent the 95%CI. HR hazard ratio, CI confidence interval, HER2 human epidermal growth factor receptor 2, TNBC triple-negative breast cancer.

1892 patients with a pCR and non-missing risk factors were included in multivariate Cox regression analyses of potential risk factors. In the entire cohort, DFS, DDFS, and OS were significantly worse for patients with a positive cN status at baseline (cN+ vs cN0 hazard ratio 1.94, 95%CI 1.48–2.54, *p* < 0.001 for DFS; hazard ratio 2.29, 95%CI 1.64–3.19, *p* < 0.001 for DDFS; hazard ratio 1.98, 95%CI 1.31–2.98, *p* = 0.001 for OS) (Fig. 1a–c). A worse DDFS and OS was seen in patients with lobular tumor type (lobular vs other tumor type hazard ratio 1.95, 95% CI 1.02–3.70, *p* = 0.043 for DDFS; hazard ratio 2.47, 95%CI 1.19–5.10, *p* = 0.015 for OS) (Fig 1b, c). The results for the various biological subtypes were inconsistent for DFS, DDFS, and OS (Fig. 1a–c). Patients with TNBC patients and patients with HER2 + /HR- tumors had a significantly shorter DFS compared to the rest (TNBC vs HER2-/HR + hazard ratio 1.49, 95%CI 1.03–2.17, *p* = 0.036 and HER2 + /HR- vs HER2-/HR + hazard ratio 1.61, 95%CI 1.06–2.45, *p* = 0.027). OS was significantly worse in TNBC patients compared to HR + /HER2- patients (TNBC vs HER2-/HR + hazard ratio 1.84, 95%CI 1.06–3.21, *p* = 0.031).

### Potential risk factors and outcome in TNBC

**Fig. 2:**
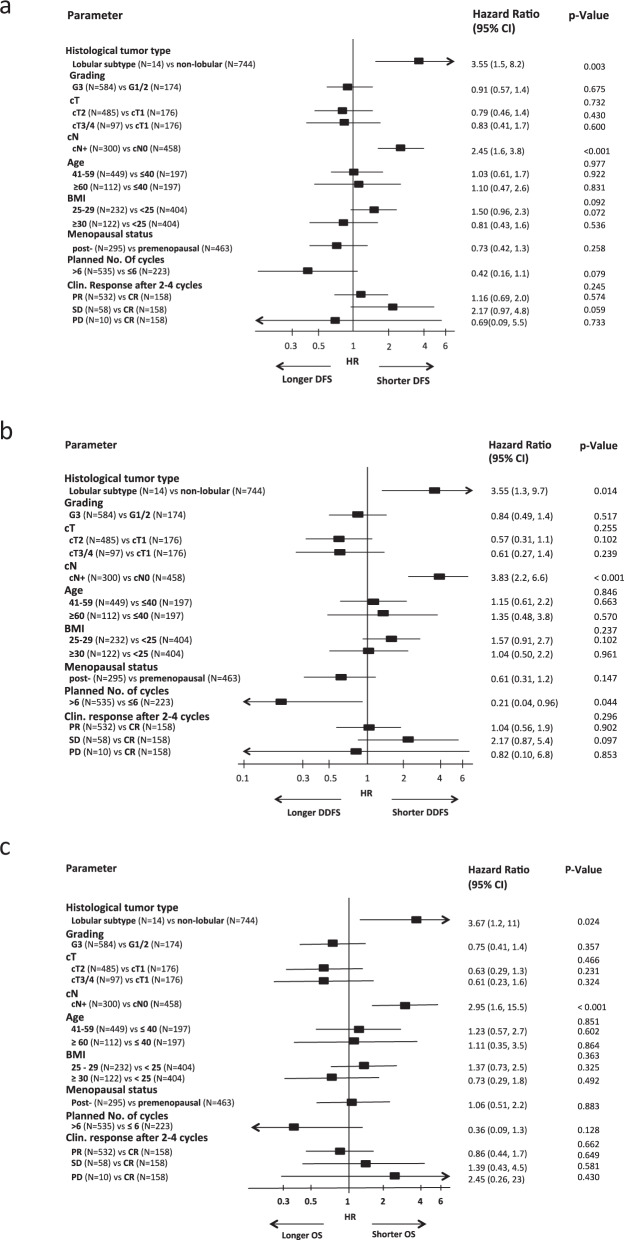
Multivariate Cox regression models for disease-free survival (a), distant disease-free survival (b) and overall survival (c) in TNBC cohort. Error bars represent the 95%CI. HR hazard ratio, CI confidence interval, HER2 human epidermal growth factor receptor 2, TNBC triple-negative breast cancer.

Multivariate Cox regression analyses in biological tumor subtypes revealed that TNBC patients with a pCR were at higher risk for a DFS, DDFS, and OS event in case of lobular histology (DFS hazard ratio 3.55, 95%CI 1.53–8.23, *p* = 0.003, DDFS hazard ratio 3.55, 95%CI 1.30–9.73, *p* = 0.014 and OS hazard ratio 3.67, 95%CI 1.19–11.4, *p* = 0.024). Clinically positive lymph nodes at baseline were also associated with a higher risk for a shorter DFS, DDFS, and OS (DFS hazard ratio 2.45, 95%CI 1.59–3.79, *p* < 0.001; DDFS hazard ratio 3.83, 95%CI 2.22–6.61, *p* < 0.001 and OS hazard ratio 2.95, 95%CI 1.58–5.50, *p* < 0.001) (Fig. 2a–c). Interestingly, patients with >6 scheduled cycles of neoadjuvant treatment had a longer DDFS (hazard ratio 0.21, 95%CI 0.04–0.96, *p* = 0.044) (Fig. 2b) compared to patients with a shorter therapy.

### Potential risk factors and outcome in patients with HER2 + disease

**Fig. 3:**
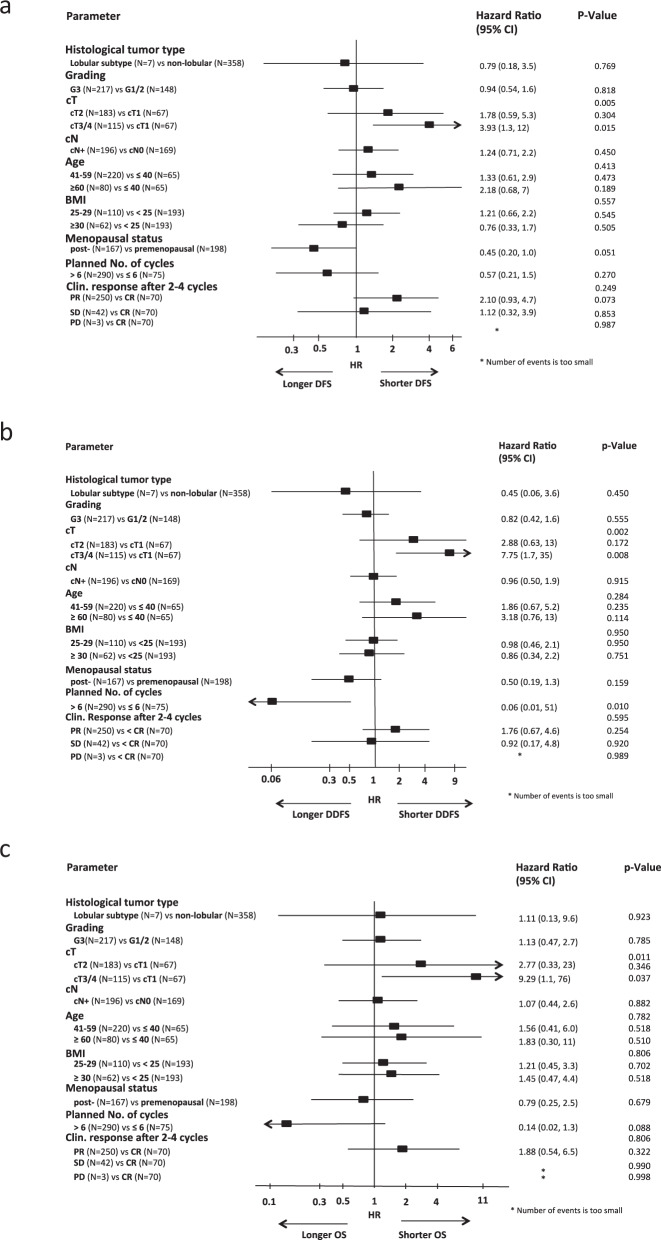
Multivariate Cox regression models for disease-free survival (a), distant disease-free survival (b) and overall survival (c) in HER2 + /HR- cohort. Error bars represent the 95%CI. HR hazard ratio, CI confidence interval, HER2 human epidermal growth factor receptor 2, TNBC triple-negative breast cancer.

Patients with HER2-positive cT3/4 tumors were at a significantly higher risk for a DFS, DDFS, and OS event compared to HER2-positive cT1 tumors (DFS hazard ratio 2.07, 95%CI 1.06–4.03, *p* = 0.033; DDFS hazard ratio 3.28, 95%CI 1.38–7.83, *p* = 0.007; OS hazard ratio 3.42, 95%CI 1.08–10.8, *p* = 0.036; multivariate Cox regression analyses). This effect was mainly seen in the HER2 + /HR- subtype (DFS hazard ratio 3.93, 95%CI 1.30–11.8, *p* = 0.015; DDFS: hazard ratio 7.75, 95%CI 1.70–35.2, *p* = 0.008; OS: hazard ratio 9.29, 95%CI 1.14–75.7, *p* = 0.037; multivariate Cox regression analyses) (Fig. 3a–c). The risk for a DDFS event was lower in patients with HER2 + /HR- tumors receiving >6 cycles of planned treatment (hazard ratio 0.06, 95%CI 0.01–0.51; *p* = 0.010; multivariate Cox regression analyses). This was not the case for DFS or OS.

**Fig. 4:**
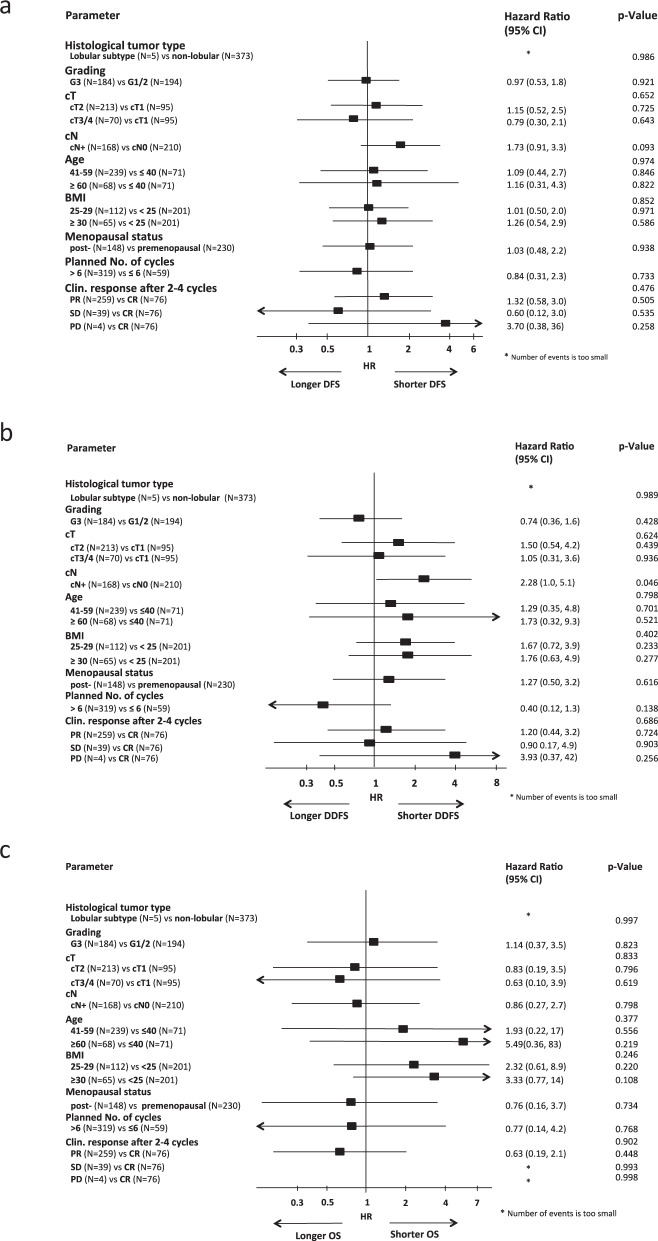
Multivariate Cox regression models for disease-free survival (a), distant disease-free survival (b) and overall survival (c) in HER2 + /HR + cohort. Error bars represent the 95%CI. HR hazard ratio, CI confidence interval, HER2 human epidermal growth factor receptor 2, TNBC triple-negative breast cancer.

In the subgroup of HER2 + /HR + breast cancer, none of the potential risk factors significantly predisposed patients to a DFS or OS event (Fig. 4a–c). Only a positive nodal status at baseline significantly increased the risk for a DDFS event in all pts (hazard ratio 2.28, 95%CI 1.02–5.12, *p* = 0.046; multivariate Cox regression analyses).

In the subgroup of HER2-/HR + breast cancer receiving neoadjuvant chemotherapy, pCR patients with cN+ at baseline had a higher risk for a DFS event than cN0 patients (hazard ratio 2.24, 95%CI 1.18–4.25, *p* = 0.013; multivariate Cox regression analyses) but none of the other potential risk factors significantly increased the risk for a DDFS or OS event.

### 4-year DFS, DDFS, and OS rates overall and in subgroups

The 4-year DFS, DDFS, and OS rates are presented in Table 2. At 4 years DFS rates were 91.9% for HER2-/HR + , 90.2% for TNBC, 89.0% for HER2 + /HR + and 87.9% for HER2 + /HR- patients. Lobular compared to other histological subtypes was associated with worse DFS, DDFS, and OS rates. Lower DFS, DDFS, and OS rates at 4 years were also detected for cT3/4 tumors compared to cT1 or cT2 tumors and for cN+ compared to cN0 status at baseline. 4-year survival rates of breast cancer subtypes according to HER2 and HR status are presented in detail in Table 3. Of clinical interest, the risk of having a DFS event within 4 years was > 10% in HER2 + patients with cT3/4 tumors or nodal involvement at primary diagnosis.

**Table 2:** Four-year rates for DFS, DDFS, and OS according to subgroups.

	DFS rate (95% CI)	DDFS rate (95% CI)	OS rate (95% CI)
Overall	89.6% (88.1%, 90.9%)	92.5% (91.2%, 93.6%)	95.2% (94.2%, 96.1%)
Subgroups			
cT1	91.2% (87.9%, 93.6%)	93.9% (91.0%, 95.8%)	96.1% (93.7%, 97.7%)
cT2	90.2% (88.3%, 91.8%)	93.4% (91.7%, 94.7%)	96.0% (94.6%, 97.0%)
cT3/4	85.7% (81.5%, 89.0%)	88.0% (84.1%, 91.0%)	91.8% (88.4%, 94.3%)
cN0	92.2% (90.4%, 93.7%)	95.0% (93.4%, 96.2%)	96.9% (95.6%, 97.8%)
cN+	86.5% (83.9%, 88.6%)	89.5% (87.3%, 91.4%)	93.3% (91.4%, 94.8%)
Lobular tumor type	82.6% (70.7%, 90.0%)	87.4% (76.4%, 93.5%)	90.6% (80.3%, 95.7%)
Non-lobular tumor type	89.8% (88.3%, 91.1%)	92.6% (91.3%, 93.8%)	95.4% (94.3%, 96.3%)
HER2-/HR +	91.9% (88.6%, 94.3%)	94.7%(91.8%, 96.5%)	97.0%(94.7%, 98.3%)
TNBC	90.2% (87.8%, 92.2%)	92.9%(90.8%, 94.5%)	94.6%(92.6%, 96.0%)
HER2 + /HR +	89.0% (85.2%, 91.8%)	91.4%(88.0%, 93.9%)	96.8%(94.3%, 98.2%)
HER2 + /HR-	87.9% (84.0%, 90.9%)	91.2%(87.6%, 93.7%)	94.2%(91.2%, 96.2%)
Ki-67 ≤ 20%	89.9%(85.0%, 93.2%)	91.9%(87.4%, 94.8%)	96.5%(93.1%, 98.2%)
Ki-67 > 20%	89.9%(87.8%, 91.7%)	92.9%(91.1%, 94.4%)	95.5%(93.9%, 96.7%)
Grade 1/2	88.1%(85.4%, 90.3%)	91.0%(88.6%, 92.9%)	95.1%(93.2%, 96.5%)
Grade 3	90.6%(88.7%, 92.2%)	93.1%(91.5%, 94.4%)	95.2%(93.7%, 96.3%)
Age ≤ 40 years	90.1%(86.9%, 92.6%)	93.6%(90.9%, 95.5%)	95.6%(93.3%, 97.2%)
Age 41–59 years	89.1%(87.1%, 90.8%)	92.1%(90.3%, 93.5%)	95.2%(93.8%, 96.3%)
Age ≥ 60 years	90.8%(86.9%, 93.5%)	92.4%(88.8%, 94.9%)	94.7%(91.5%, 96.7%)
BMI < 25	90.4%(88.3%, 92.1%)	94.0%(92.3%, 95.3%)	95.9%(94.5%, 97.0%)
BMI 25–29	87.7%(84.7%, 90.1%)	89.9%(87.1%, 92.1%)	94.2%(91.9%, 95.8%)
BMI ≥ 30	90.7%(86.8%, 93.5%)	92.5%(88.9%, 94.9%)	95.0%(91.8%, 97.0%)
Premenopausal	88.7%(86.7%, 90.4%)	92.3%(90.6%, 93.8%)	95.3%(93.9%, 96.4%)
Postmenopausal	90.8%(88.5%, 92.7%)	92.6%(90.5%, 94.3%)	95.1%(93.3%, 96.5%)
≤6 planned CT cycles	89.1%(85.7%, 91.8%)	91.5%(88.4%, 93.9%)	93.7%(90.8%, 95.7%)
>6 planned CT cycles	89.7%(88.1%, 91.2%)	92.8%(91.3%, 94.0%)	95.7%(94.5%, 96.6%)
Complete response after 2–4 cycles	91.7%(88.4%, 94.2%)	93.4%(90.4%, 95.6%)	94.2%(91.3%, 96.2%)
Partial response after 2–4 cycles	89.0%(87.1%, 90.6%)	92.2%(90.6%, 93.5%)	95.1%(93.8%, 96.2%)
Stable disease after 2–4 cycles	90.0% (84.1%, 93.8%)	93.3%(88.2%, 96.2%)	97.5% (93.5%, 99.1%)
Progress after 2–4 cycles	84.7%(59.7%, 94.8%)	84.1%(58.3%, 94.6%)	94.7%(68.1%, 99.2%)
ypT0	90.8% (89.2%, 92.2%)	93.0% (91.6%, 94.1%)	95.4% (94.2%, 96.3%)
ypTis	84.5% (80.3%, 87.9%)	90.5% (86.9%, 93.1%)	94.8% (91.8%, 96.7%)

*BMI* body mass index, *DDFS* distant disease-free survival, *DFS* disease-free survival, *ER* estrogen receptor, *PgR* progesterone receptor, *HER2* human epidermal growth factor receptor 2, *HR* hormone receptor, *OS* overall survival, *TNBC* triple-negative breast cancer

**Table 3:** Four-year rates for DFS, DDFS, and OS in subgroups by biological subtype.

	HER2-/HR +			TNBC			HER2 + /HR +			HER2 + /HR-		
Subgroups	DFS rate (95% CI)	DDFS rate (95% CI)	OS rate (95% CI)	DFS rate (95% CI)	DDFS rate (95% CI)	OS rate (95% CI)	DFS rate (95% CI)	DDFS rate (95% CI)	OS rate (95% CI)	DFS rate (95% CI)	DDFS rate (95% CI)	OS rate (95% CI)
cT1	92.7% (83.4%, 96.9%)	95.6% (86.9%, 98.6%)	98.6% (90.4%, 99.8%)	87.8% (81.7%, 92.0%)	91.1% (85.6%, 94.5%)	93.8% (88.8%, 96.6%)	92.3% (84.6%, 96.3%)	94.5% (87.2%, 97.7%)	96.7% (90.3%, 98.9%)	97.0% (88.4%, 99.2%)	98.5% (89.6%, 99.8%)	98.4% (89.4%, 99.8%)
cT2	91.6% (87.3%, 94.5%)	94.6% (90.9%, 96.8%)	97.1% (94.0%, 98.6%)	91.3% (88.4%, 93.6%)	94.2% (91.6%, 96.0%)	95.6% (93.3%, 97.2%)	88.5% (83.1%, 92.2%)	91.5% (86.7%, 94.7%)	96.8% (92.9%, 98.5%)	89.7% (84.2%, 93.4%)	93.2% (88.4%, 96.1%)	96.6% (92.5%, 98.4%)
cT3/4	92.1% (82.0%, 96.6%)	93.8% (84.3%, 97.6%)	95.3% (86.0%, 98.4%)	88.7% (80.6%, 93.6%)	89.7% (81.6%, 94.3%)	90.6% (82.7%, 95.0%)	85.8% (75.2%, 92.1%)	87.2% (76.8%, 93.1%)	96.9% (88.0%, 99.2%)	79.3% (70.2%, 85.9%)	83.2% (74.5%, 89.1%)	87.6% (79.6%, 92.6%)
cN0	93.9% (89.5%, 96.5%)	95.9% (92.0%, 97.9%)	99.0% (96.0%, 99.7%)	93.3% (90.4%, 95.3%)	96.1% (93.8%, 97.6%)	96.7% (94.6%, 98.1%)	92.9% (88.2%, 95.7%)	96.1% (92.3%, 98.0%)	97.5% (94.2%, 99.0%)	88.4% (82.2%, 92.6%)	91.0% (85.2%, 94.6%)	95.6% (91.1%, 97.9%)
cN+	89.5% (83.8%, 93.3%)	93.1% (88.1%, 96.0%)	94.7% (90.1%, 97.2%)	85.6% (81.0%, 89.2%)	88.0% (83.6%, 91.3%)	91.5% (87.5%, 94.2%)	84.5% (77.9%, 89.3%)	86.1% (79.6%, 90.7%)	95.7% (90.7%, 98.1%)	87.4% (81.8%, 91.4%)	91.3% (86.3%, 94.5%)	93.0% (88.3%, 95.9%)
Lobular tumor type	83.6% (64.9%, 92.8%)	87.1% (69.1%, 94.9%)	93.5% (76.5%, 98.3%)	70.1% (42.3%, 86.3%)	82.4% (54.7%, 93.9%)	82.4% (54.7%, 93.9%)	100% (100%, 100%)	100% (100%, 100%)	100% (100%, 100%)	87.5% (38.7%, 98.1%)	87.5% (38.7%, 98.1%)	87.5% (38.7%, 98.1%)
Non-lobular tumor type	92.7% (89.3%, 95.0%)	95.3% (92.5%, 97.1%)	97.3% (94.9%, 98.6%)	90.7% (88.3%, 92.6%)	93.1% (91.0%, 94.8%)	94.8% (92.9%, 96.3%)	88.8% (85.0%, 91.6%)	91.3% (87.8%, 93.8%)	96.7% (94.1%, 98.2%)	87.9% (83.9%, 91.0%)	91.2% (87.7%, 93.8%)	94.4% (91.3%, 96.4%)
Ki-67 ≤ 20%	92.0% (80.0%, 96.9%)	96.0% (84.9%, 99.0%)	98.0% (86.6%, 99.7%)	90.9% (68.1%, 97.7%)	90.9% (68.1%, 97.7%)	96.0% (74.8%, 99.4%)	87.8% (78.4%, 93.3%)	89.4% (80.5%, 94.3%)	97.6% (90.8%, 99.4%)	90.5% (80.0%, 95.6%)	92.2% (82.1%, 96.7%)	94.0% (84.7%, 97.7%)
Ki-67 > 20%	91.3% (85.5%, 94.9%)	92.0% (86.3%, 95.4%)	95.1% (89.9%, 97.6%)	90.3% (86.9%, 92.8%)	93.8% (90.9%, 95.8%)	95.7% (93.1%, 97.3%)	90.8% (85.8%, 94.1%)	94.4% (90.0%, 96.8%)	97.4% (93.8%, 98.9%)	86.7% (80.6%, 91.0%)	90.3% (84.8%, 93.8%)	93.6% (88.8%, 96.4%)
Grade 1/2	90.8% (85.6%, 94.2%)	94.1% (89.6%, 96.7%)	97.3% (93.7%, 98.9%)	87.1% (80.9%, 91.4%)	89.7% (83.9%, 93.5%)	92.5% (87.1%, 95.7%)	87.0% (81.3%, 91.1%)	89.6% (84.2%, 93.3%)	96.5% (92.4%, 98.4%)	87.0% (80.1%, 91.6%)	90.6% (84.3%, 94.4%)	94.1% (88.5%, 97.0%)
Grade 3	93.2% (88.4%, 96.1%)	94.9% (90.4%, 97.3%)	96.5% (92.3%, 98.4%)	91.2% (88.5%, 93.3%)	93.8% (91.4%, 95.5%)	95.1% (92.9%, 96.7%)	90.5% (85.0%, 94.1%)	93.0% (88.0%, 96.0%)	97.0% (92.8%, 98.7%)	88.4% (83.2%, 92.1%)	91.4% (86.7%, 94.5%)	94.3% (90.1%, 96.7%)
Age ≤ 40 years	94.6% (87.6%, 97.7%)	96.7% (90.2%, 98.9%)	98.9% (92.7%, 99.8%)	90.1% (84.9%, 93.6%)	92.8% (88.2%, 95.7%)	94.8% (90.6%, 97.2%)	91.0% (80.9%, 95.9%)	95.4% (86.3%, 98.5%)	98.2% (87.8%, 99.7%)	88.3% (77.0%, 94.3%)	93.2% (82.9%, 97.4%)	94.9% (84.9%, 98.3%)
Age 41–59 years	89.9% (85.1%, 93.1%)	93.1% (88.9%, 95.7%)	96.0% (92.5%, 97.9%)	90.8% (87.6%, 93.1%)	93.1% (90.2%, 95.1%)	94.6% (92.0%, 96.4%)	88.0% (83.0%, 91.6%)	90.7% (86.1%, 93.9%)	97.2% (93.9%, 98.8%)	86.9% (81.5%, 90.7%)	90.8% (86.0%, 93.9%)	94.3% (90.2%, 96.7%)
Age ≥ 60 years	96.1% (85.2%, 99.0%)	98.0% (86.9%, 99.7%)	98.0% (86.9%, 99.7%)	88.4% (80.4%, 93.2%)	92.3% (85.1%, 96.1%)	94.1% (87.2%, 97.3%)	89.8% (79.6%, 95.0%)	89.8% (79.6%, 95.0%)	94.0% (84.7%, 97.7%)	90.6% (81.3%, 95.4%)	90.6% (81.3%, 95.4%)	93.3% (84.8%, 97.2%)
Premenopausal	92.6% (88.4%, 95.4%)	95.2% (91.5%, 97.3%)	97.8% (94.9%, 99.1%)	89.6% (86.3%, 92.1%)	92.6% (89.8%, 94.7%)	94.7% (92.2%, 96.5%)	89.0% (84.1%, 92.5%)	92.7% (88.3%, 95.5%)	97.0% (93.4%, 98.7%)	84.4% (78.3%, 88.9%)	89.9% (84.5%, 93.4%)	94.0% (89.3%, 96.6%)
Postmenopausal	90.7% (84.5%, 94.5%)	93.8% (88.3%, 96.7%)	95.7% (90.6%, 98.0%)	91.1% (87.1%, 93.9%)	93.2% (89.5%, 95.6%)	94.2% (90.7%, 96.4%)	88.8% (82.3%, 93.0%)	89.5% (83.2%, 93.5%)	96.4% (91.5%, 98.5%)	91.9% (86.4%, 95.2%)	92.5% (87.2%, 95.7%)	94.4% (89.5%, 97.0%)
ypT0	91.7% (87.8%, 94.4%)	94.7% (91.3%, 96.8%)	97.2% (94.4%, 98.6%)	91.1% (88.6%, 93.1%)	93.2% (91.0%, 94.9%)	95.0% (93.0%, 96.4%)	91.0% (87.0%, 93.9%)	92.7% (88.9%, 95.2%)	96.6% (93.5%, 98.2%)	90.6% (86.4%, 93.5%)	92.1% (88.1%, 94.8%)	94.6% (91.1%, 96.8%)
ypTis	92.4% (84.7%, 96.3%)	94.6% (87.5%, 97.7%)	96.7% (90.0%, 98.9%)	83.8% (74.2%, 90.1%)	90.6% (82.1%, 95.2%)	91.6% (83.0%, 95.9%)	82.0% (71.9%, 88.8%)	87.0% (77.8%, 92.6%)	97.4% (90.1%, 99.4%)	79.7% (69.2%, 86.9%)	88.2% (79.1%, 93.5%)	93.1% (85.4%, 96.9%)

*BMI* body mass index, *DDFS* distant disease-free survival, *DFS* disease-free survival, *ER* estrogen receptor, *PgR* progesterone receptor, *HER2* human epidermal growth factor receptor 2, *HR* hormone receptor, *OS* overall survival, *TNBC* triple-negative breast cancer

## Discussion

This analysis identified potential clinical risk factors in women with a pCR after neoadjuvant chemotherapy combined with anti-HER2 therapy in cases of HER2-positive disease. Overall, nodal involvement at diagnosis,cT3/4 tumors as well as lobular histology were identified being the most adverse factors in patients with a pCR, underlining that tumor burden at the time of diagnosis is important. In patients with TNBC, an initial positive nodal status and lobular histology were predicting a significantly higher risk of relapse or death. In patients with HER2-positive disease tumors initially classified as cT3/4 tumors indicated a higher risk of relapse and death, again very consistently for DFS, DDFS, and OS. This effect was strongest in the HER2-positive hormone receptor-negative subgroup. Four-year DFS and DDFS rates for patients included in this pooled analysis were lowest in those patients with lobular histology, cN+, and cT3/4 tumors.

Several individual trials and meta-analyses showed that a pCR following neoadjuvant chemotherapy improves long-term outcomes in terms of DFS, DDFS, and OS and this was in particular seen in patients with more aggressive tumor types like triple-negative or HER2-positive tumors^3–5,22^. However, up to 20% of patients with pCR will eventually relapse.

In individual studies long-term outcome is mainly driven by pCR vs no pCR, but also tumor size and nodal involvement assessed before neoadjuvant therapy resulted in an inferior outcome^23,24^. This is also reflected by the fact that these factors are included in the clinical pathological stage (CPS) score with poorer outcome in case of a more advanced tumor extent in HR positive BC^25–27^. However, none of these studies analyzed data of patients with and without pCR separately due to limitations in the number of patients and events. Several neoadjuvant trials have shown that patients with small tumors or no nodal involvement at diagnosis are more likely to have a pCR^23,28,29^. It is of interest that in patients diagnosed with a more advanced clinical stage before neoadjuvant treatment a pCR was less likely and in case a pCR was present the relapse rates were still higher compared to those patients with a pCR and initially small tumors or negative nodes. An explanation might be that these patients may have more systemic disseminated tumor cells at diagnosis, which are resistant to treatment. In fact, trials investigating the role of circulating tumor cells (CTCs) in neoadjuvantly treated patients demonstrated that the presence of CTCs before and not after neoadjuvant therapy negatively influenced the outcome in terms of OS, DDFS, and locoregional relapse-free survival^30,31^. The number of CTCs before the start of neoadjuvant therapy was also important with the worst outcome seen in patients with the highest CTC counts. In the meta-analysis of Bidard et al., the detection of CTCs at diagnosis was significantly associated with greater tumor size^30^. A retrospective analysis on tissues from the primary and the metastases from patients with a recurrent disease despite pCR and relapse matched to controls of pts with a pCR but no relapse showed the potential of transcriptomic analyses in this understudied cohort^32^.

Earlier analyses demonstrated that patients with lobular subtypes have lower pCR rates after neoadjuvant chemotherapy compared with invasive ductal carcinomas^33^. However, if they had no pCR their outcome was better compared to the non-lobular types. The low responses of lobular histology can be explained at least in part by their particular biologic profile with low proliferation rates, positive hormone receptors, and low grade^34^. Thus, patients with pure lobular histology (G2 and HR + /HER2-) are usually not candidates for the use of neoadjuvant chemotherapy and in fact only 3–4% of the patients included in the five GBG neoadjuvant trials were diagnosed with an invasive lobular subtype. From the GeparSepto study onwards patients with tumors of pure lobular histology were excluded from trial participation. However, there are also subtypes of lobular histology being high grade, hormone receptor-negative, and HER2-positive and in those lobular tumors with more aggressive biological features pCR rates were significantly higher with up to 20%^33^. In our investigation relapse rates were significantly higher in the non-classical lobular cohort (TNBC or HER2-positive) which may confirm that this histologic subtype is different from the pure lobular cancers. These high-risk lobular types may be enriched with HER2 mutations^35,36^.

A pooled analysis has several limitations. One of them is that the treatment regimens were quite different since the included trials were conducted between 2002 and 2013. However, the chemotherapy backbone in every trial was an anthracycline-taxane-based regimen, which can still be considered the standard of care, except for the paclitaxel and liposomal doxorubicin treatment applied in the GeparSixto trial. However, as this study focuses on patients with a pCR after neoadjuvant treatment, it was considered justifiable to also include patients from this trial as an anthracycline was administered. Taking the chemotherapy backbone in consideration, the findings may not be extrapolated to patients achieving a pCR with different treatment regimens. The limitation could only be overcome by conducting this analysis in patients that were treated with the same chemotherapy regimen. As relapses in breast cancer can frequently be seen beyond 5 years, especially in those of HR + subtype, our median follow-up time of nearly 5 years may be still too short. However, our trial population was enriched with biologically aggressive tumors, where typically most of the relapses occur during the first 5 years^37,38^, therefore our follow-up time most likely covers the majority of relapses. To overcome this limitation a longer follow-up would be necessary and this would implement that the analysis should be repeated in the future to confirm the conclusions. The strength of our analysis is that we could include almost 2,000 patients with a pCR in this pooled analysis. Our analysis was based on five prospectively randomized phase 3 neoadjuvant trials of a study collaboration (GBG/AGO-B), ensuring a very homogenous assessment of all study procedures like pre-treatment tumor assessments, central review of histopathological reports, and definition of pCR but also of additional study procedures including further post-surgical treatments which are usually not part of neoadjuvant trials. Furthermore, lymph node status before treatment was consistently assessed throughout all trials not only clinically but also with ultrasound.

Taken together this is a pooled analysis identifying factors predicting a relapse after pathological complete response following neoadjuvant chemotherapy. In our analysis lobular histology, larger tumor size and initially involved lymph nodes indicated a higher risk for DFS, DDFS, and OS events after a pCR following neoadjuvant treatment. The importance of these risk factors varied by intrinsic subtype, with nodal status and lobular histology as predictors in the triple-negative cohort, and the tumor size in the HER2-positive cohort. Thus, factors predicting a higher risk of relapse after a pCR were tumor extent before therapy and histological type. These data might become of clinical relevance, especially after validation, as treatments are available now for patients at high risk after neoadjuvant therapy, namely those with no pCR, i.e. T-DM1 for HER2 + , capecitabine, and pembrolizumab for TNBC and most recently, PARP inhibitors for patients with *BRCA*1/2 mutations and CDK 4/6 inhibitors for HER2-/HR + patients. So far, most trials recruiting in the postneoadjuvant setting focus on patients that did not experience a pCR. Taking the results of the here presented analysis into consideration there is also a group of patients with pCR that have a higher risk of relapse and should therefore be considered for an additional treatment despite pCR in postneoadjuvant studies to improve long-term outcome of this otherwise neglected patient population.

## Patients and methods

### Patients

Data from the five neoadjuvant trials GeparTrio (NCT00544765) (ethics committee of the University of Frankfurt), GeparQuattro (NCT00288002) (ethics committee of the University of Frankfurt), GeparQuinto (NCT00567554) (ethics committee of the University of Frankfurt), GeparSixto (NCT01426880) (ethics committee Nordrhein, Duesseldorf) and GeparSepto (NCT01583426) (ethics committee Berlin) conducted between 2002 and 2013 were pooled and only patients with a pCR (defined as no microscopic evidence of residual invasive tumor cells in any resected specimens of the breast and axillary nodes with in-situ residuals being allowed (ypT0/ypTis, ypN0)) were considered. Individual results and study designs of these studies have previously been reported^8–21^. All trials were approved by the respective ethics committees and patients had given written informed consent for study participation and data collection. All trials had comparable main eligibility criteria and used an anthracycline-taxane-based chemotherapy backbone (Supplementary table 1). The GeparSixto study enrolled only patients with triple-negative and HER2-positive breast cancer. Patients with HER2-positive disease received anti-HER2 treatment as part of their neoadjuvant therapy within the GeparQuattro, GeparQuinto, GeparSixto, and GeparSepto study. In the GeparTrio trial patients with HER2-positive tumors did not receive any anti-HER2 therapy as part of their neoadjuvant or adjuvant treatment since this was the clinical practice when the study was conducted between 2002 and 2005. Patients with HER2-positive disease from GeparTrio were therefore excluded from the analysis. After surgery, anti-HER2 therapy, endocrine therapy in patients with hormone receptor-positive breast cancer, and radiotherapy were given according to current national guidelines. The tumor subtype was centrally tested.

### Objectives and endpoints

The primary objective of this pooled analysis was to characterize patients at higher risk for relapse despite a pCR after neoadjuvant chemotherapy for early breast cancer. Therefore, the influence of predefined potential risk factors as biological subtype (HER2-negative/Hormone receptor (HR)-positive, TNBC, HER2-positive/HR-positive, HER2-positive/HR negative), histological tumor type (lobular subtype, other), tumor grade (G1/G2, G3), tumor stage at baseline (cT1, cT2, cT3/4), nodal stage at baseline (cN0, cN + ), age (≤40, 41–59, ≥ 60 years), BMI ( < 25, 25–29, ≥ 30), menopausal status (pre-, postmenopausal), scheduled number of chemotherapy cycles (≤6, >6) and clinical response after 2–4 cycles (stable disease, partial response, complete response, progressive disease) on DFS was analysed. Secondary objectives were to assess the influence of the same risk factors on distant disease-free survival (DDFS) and overall survival (OS). Ki-67 (≤20% vs higher) was considered as a covariate in preliminary analyses but was excluded due to too many missing values in GeparTrio, GeparQuattro, and GeparQuinto where Ki-67 was not centrally assessed.

DFS was defined as the time in months from randomization to first relapse (local or distant), secondary malignancy, or death from any cause, whichever occurred first^39^. DDFS was defined as the time in months from randomization to any distant recurrence of disease, any secondary malignancy, or death due to any cause, whichever occurred first; OS as the time in months from randomization to death due to any cause. Patients without an event were censored at the date of the last contact.

### Statistical analysis

Multivariate (including all potential risk factors and study) Cox proportional hazards models were used to report hazard ratios with 95% confidence intervals (CIs), adjusted for study to account for possible heterogeneity between the trials. Patients with missing values in risk factors were excluded from multivariate Cox regression models. Patients with missing values for the variable defining the subgroup were excluded from the analyses in this subgroup.

For every potential risk factor, 4-year DFS, DDFS, and OS rates and the corresponding 95% CIs were estimated using the Kaplan–Meier method.

All endpoints were additionally analyzed in the subgroups according to biological subtype.

All reported *p*-values with *p* < 0.05 were considered statistically significant. No adjustment for multiple testing was performed. SAS versions 9.2 and 9.4 under SAS Enterprise Guide 4.3 and 8.5 were used to perform the analyses.

### Reporting summary

Further information on research design is available in the Nature Research Reporting Summary linked to this article.

## Supplementary information

Supplementary material

Reporting Summary

## Data Availability

Individual participant data that underlie the results reported in this article, after final analysis and publication of all secondary efficacy endpoints, study protocol and statistical report will be available upon request. All relevant data are within the paper and its supporting information files. The data underlying the results presented in the study are available from GBG. Some restrictions apply due to confidentiality of patient data. Since these data are derived from a prospective clinical trial with ongoing follow up collection there are legal and ethical restrictions to share sensitive patient related data publicly. Interested groups may use the “Cooperation Proposal Form” on https://www.gbg.de/en/research/trafo.php. Data can be requested in context of a translational research project by sending the form to trafo@gbg.de. Translational research proposals are approved by the GBG scientific boards Data can be requested in context of a translational research project by sending the form to trafo@gbg.de. Translational research proposals are approved by the GBG scientific boards.
