# Supplementary material for: Identifying breast cancer patients at risk of relapse despite pathological complete response after neoadjuvant therapy
Source: NPJ Breast Cancer. 2023 Apr 7;9:23. doi: 10.1038/s41523-023-00525-2 (PMC10082019; doi:10.1038/s41523-023-00525-2)
Supplement: Supplementary file 1 — Supplementary material [file 41523_2023_525_MOESM1_ESM.pdf]

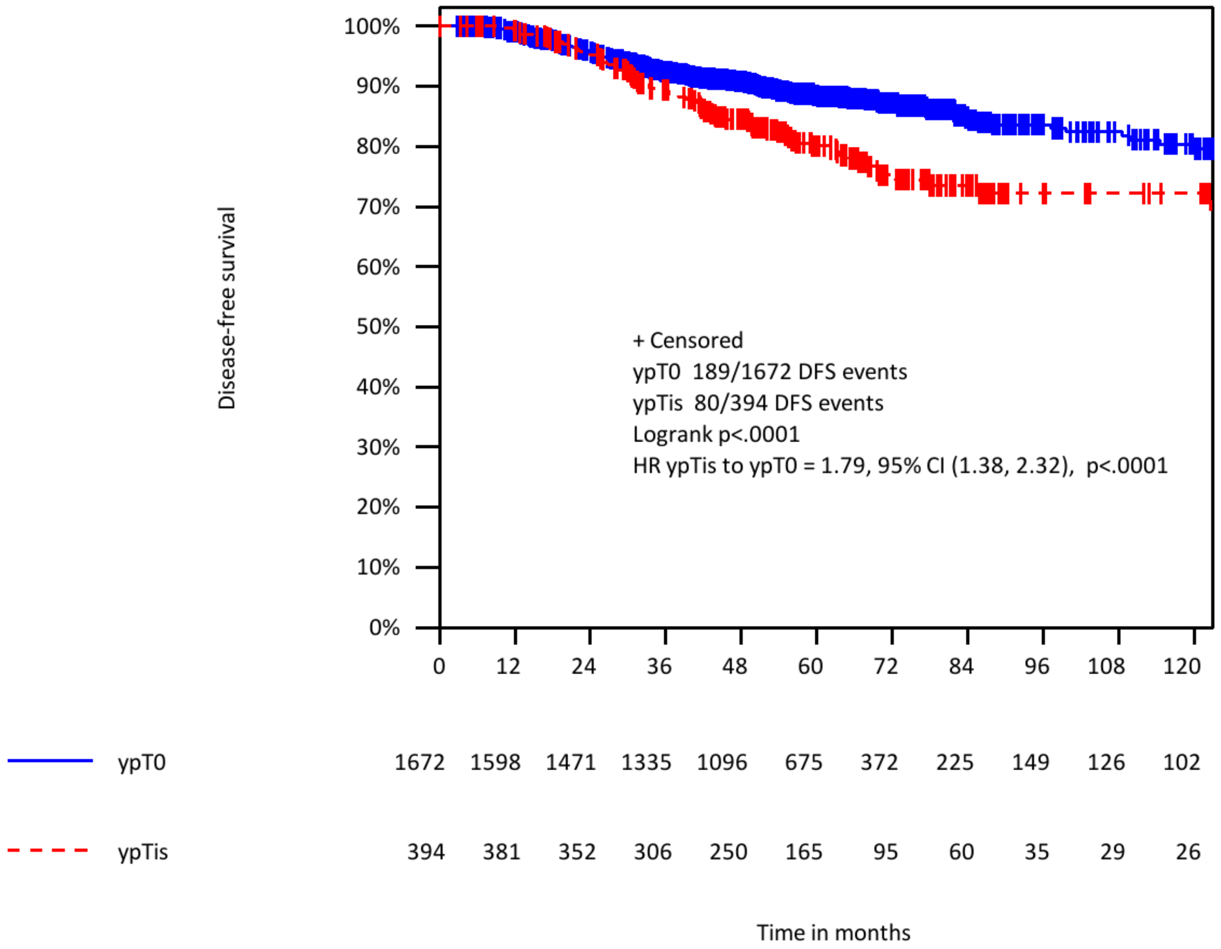

Supplementary figure 1a: Kaplan-Meier curves of disease-free survival according to residual in situ disease.

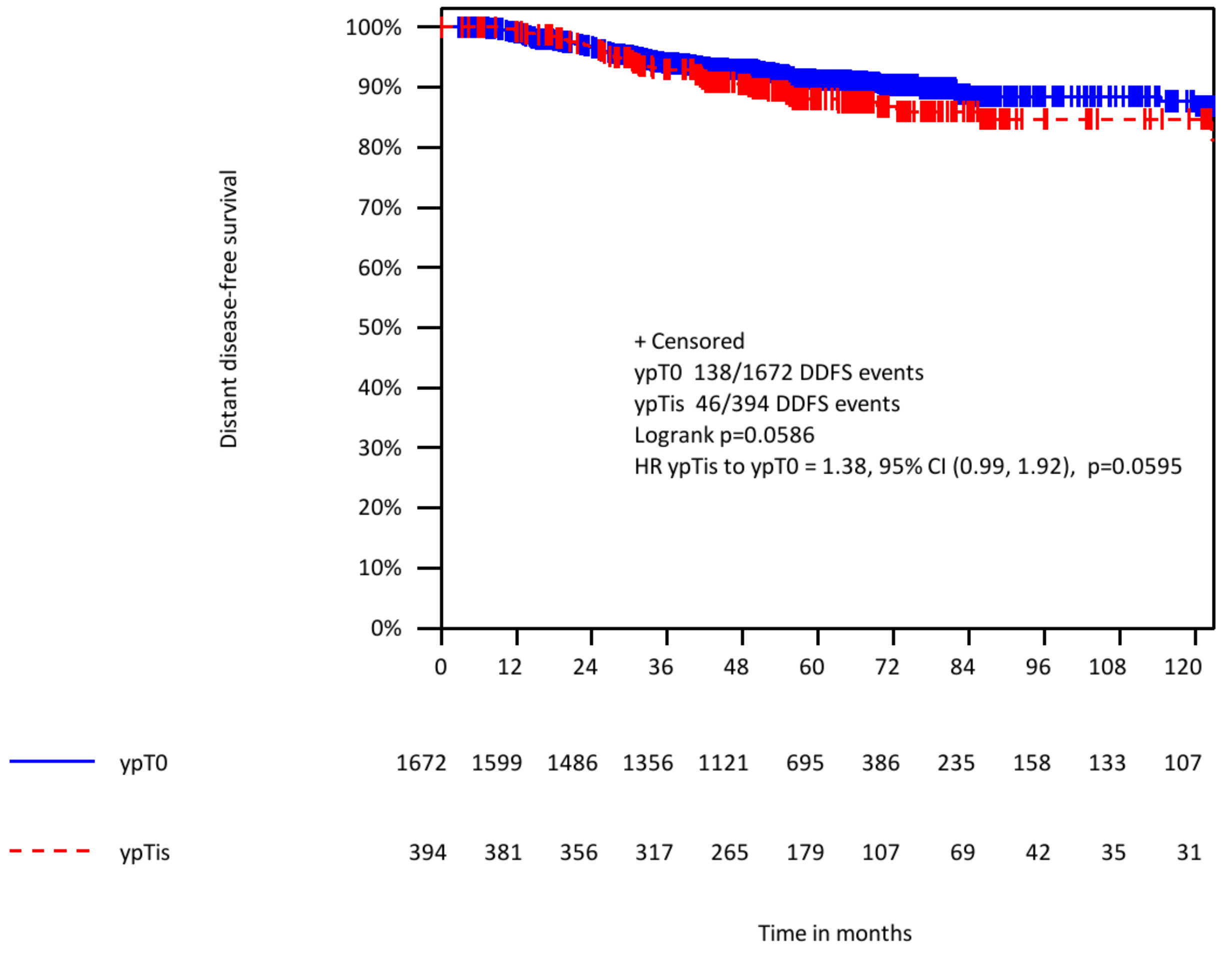

Supplementary figure 1b: Kaplan-Meier curves of distant disease-free survival according to residual in situ disease.

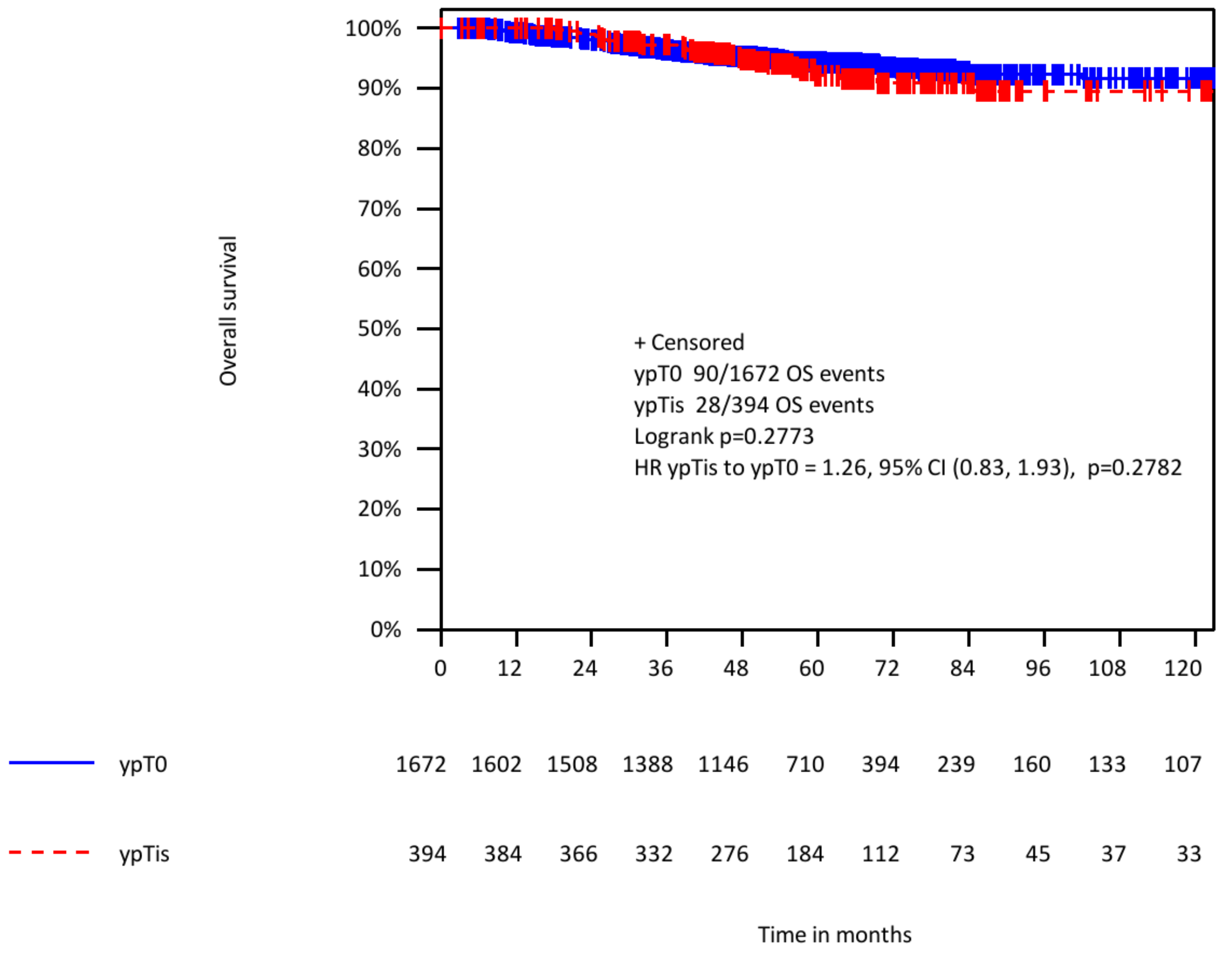

Supplementary figure 1c: Kaplan-Meier curves of overall survival according to residual in situ disease.

Supplementary table 1: Inclusion criteria and design per study

**Study**	**Inclusion criteria**	**Tumor subtypes**	**Study arms**	
			control	*experimental*
GeparTrio	High risk: <36 years, >cT2, HR-,cN+, G3	all	2*DocAC-4*DocAC	2*DocAC-NX<br>2*DocAC-6*DocAC
GeparQuattro	cT3orcT4, HR-, HR+ and cN+	all	EC-Doc	EC-DocX, EC-Doc-X
GeparQuinto	cT3/4, cT2 if HR- or cN+, cT1 if HR- or pN+	HER2+	EC-Doc+T	EC-Doc+ Lap
		HER2-	EC-Doc	EC-Doc + Bev
		Non responder after EC	EC-P	EC-P +RAD001
GeparSixto	cT2-4 or CT1c if N+	TNBC	PM+Bev	PMCb+Bev
		HER2+	PM+T+Lap	PMCb+T+Lap
GeparSepto	cT2-4, cT1c and cN+ or high risk	all	P-EC (+T+Per if HER2+)	nabP-EC (+T+Per if HER2+)

A: adriamycine; Bev: Bevacizumab; C: cyclophosphamide; Cb: carboplatinum; Doc: docetaxel; E: epirubicine; Lap: lapatinib; M: myocet; N: vinorelbine; nabP: nabPaclitaxel; P: paclitaxel; PE: pertuzumab; RAD001: everolimus; T: trastuzumab; X: capecitabine, HR: hormone receptor, TNBC: triple negative breast cancer
